# Hypobaric hypoxia aggravates neuroinflammation in ligature-induced periodontitis mice via the STAT3 signaling pathway

**DOI:** 10.3389/fimmu.2025.1600035

**Published:** 2025-07-29

**Authors:** Lu Chen, Jiayi Zhang, Xuechao Fei, Jiajing Wang, Jiayue Gao, Xiangpei Yue, Yaqun Jiang, Zibi Shi, Shaojie Zhang, Xiufang Jiang, Wenyue Chang, Zhonghua Dai, Jianjun Guo, Tong Zhao, Xiaoxia Jiang, Lingling Zhu

**Affiliations:** ^1^ Department of Brain Protection, Beijing Institute of Basic Medical Sciences, Beijing, China; ^2^ Graduate Collaborative Training Base of Beijing Institute of Basic Medical Sciences, Hengyang Medical School, University of South China, Hengyang, China; ^3^ Department of Gastroenterology, The Second Medical Center & National Clinical Research Center for Geriatric Diseases, Chinese PLA General Hospital, Beijing, China

**Keywords:** hypobaric, hypoxia, periodontitis, neuroinflammation, STAT3

## Abstract

**Background and purpose:**

Studies have shown that inflammation is a key risk factor for altitude sickness in hypobaric hypoxia environments. Periodontitis, a common oral disease, is prevalent among many individuals. This study aimed to investigate the onset and progression of neuroinflammation in mice with ligature-induced periodontitis under hypobaric hypoxia and to explore the potential underlying mechanisms.

**Methods:**

C57BL/6J mice were randomly divided into four groups: control (Con), hypobaric hypoxia (HH), periodontitis (P), and periodontitis combined with hypobaric hypoxia (PHH), which were then placed in a hypobaric hypoxia chamber for 1, 3, and 5 days. The expression of inflammatory cytokines was assessed by qPCR or ELISA. Anxiety-related behavior and memory abilities were evaluated by behavioral tests. The activation of microglia and astrocytes in the hippocampus and cortex was assessed by immunofluorescence.

**Results:**

Our results demonstrated that one day of exposure to hypobaric hypoxia in mice with periodontitis significantly exacerbated periodontal inflammation, peripheral inflammation, and neuroinflammation. Specifically, hippocampal microglia in these mice were activated following brief exposure to hypobaric hypoxia. Furthermore, the STAT3 signaling pathway was markedly activated, playing a crucial role in mediating the intensified neuroinflammation observed in periodontitis model mice subjected to one day of hypobaric hypoxia.

**Conclusion:**

Our data highlight the exacerbating effect of hypobaric hypoxia on neuroinflammation in periodontitis model mice, mediated through the activation of the STAT3 signaling pathway. These findings provide important insights and considerations for individuals with periodontitis who are planning to travel to high-altitude regions.

## 1 Introduction

As more tourists and workers travel to high-altitude regions, the risk of exposure to hypobaric hypoxia continues to rise. The impact of systemic and local inflammation on neuroinflammation following hypobaric hypoxic exposure has garnered increasing attention. Recent studies have demonstrated that hypobaric hypoxia can exacerbate cerebral inflammation in a DSS-induced colitis mouse model (1). Moreover, the combination of systemic inflammation and transient severe hypoxia has been shown to induce neuroinflammation (2, 3). These findings have established a link between hypobaric hypoxia, peripheral inflammation, local inflammation, and neuroinflammation, offering novel insights into the underlying mechanisms. Therefore, the investigation of inflammatory responses in hypobaric hypoxia environments is of critical importance and cannot be overlooked.

Periodontitis is a prevalent global infectious disease. With the synergistic effect of various factors, periodontitis resulting in irreversible consequences (4, 5). At present, the pathogenesis of periodontitis has not been fully clarified, but hypobaric hypoxia may be a risk factor (6, 7). In a survey of 309 high-altitude travelers in Nepal, 23.2% of travelers developed oral problems (8). Moreover, in a correlation analysis between gingival status and altitude in adolescents in western China, it was found that 64.09% and 77.15% of adolescents in the plateau area had gingival bleeding and dental calculus, respectively, which were significantly higher than those in the plain area (6). The above studies have shown that the incidence of periodontitis in the plateau environment is significantly higher than that in the plain area.

Moreover, a substantial body of research has established that periodontitis can induce neuroinflammation (9). The pathogenic microorganisms associated with periodontitis possess the ability to infiltrate the brain via two primary pathways: blood circulation (10) and nerve bypass (11–13). Once within the brain, these microorganisms can release virulence factors that activate immune inflammatory responses. Additionally, these periodontal pathogens release numerous virulence factors that destroy periodontal tissues (14), leading to the continuous exacerbation of local and systemic inflammation. The inflammatory cytokines generated at these sites can migrate to the brain, potentially triggering neuroinflammation and even neurodegenerative diseases (15–19). This process underscores that periodontitis is a significant risk factor for neuroinflammation. Given that periodontitis is one of the highly prevalent inflammatory diseases in high-altitude regions (6), it is imperative to investigate the impact of periodontitis on neuroinflammation under hypobaric hypoxic conditions.

In this study, we aimed to investigate the effects of hypobaric hypoxia exposure on the neuroinflammation of periodontitis model mice.

## 2 Materials and methods

### 2.1 Study design and animals

Male C57BL/6J mice, weighing (20 ± 2) g each and 8 weeks old, were purchased from the Laboratory Animal Center of SPF Technology Company in Beijing, China. All laboratory animal management and operation followed the Laboratory Animal Management Regulations of Beijing Institute of Basic Medical Sciences. The ethical review committee of animal experimental institutions approved all experimental protocols (IACUC-DWZX-2023-531). Mice were separated into four groups: normal mice group (Con), hypobaric hypoxia exposure group (HH), periodontitis group (P), and periodontitis plus hypobaric hypoxia exposure group (PHH).A periodontitis mouse model was established by ligating between the roots of the left maxillary first and second molars using silk ligation material, 5–0 surgical suture (Holycon Medical Equipment Co., Ltd., Nantong, China), as previously described (20). Prior to surgery, anesthesia was induced with 1.25% Tribromoethanol. The hypobaric hypoxia groups were placed in a hypobaric hypoxic chamber (Guizhou Feng lei Aviation Ordnance Co., Ltd., model DYC-DWI) and exposed continuously to simulated high-altitude conditions at 6000 m (369.4 mm Hg, 10.16% O_2_). The chamber temperature was maintained at 22°C -26°C, with humidity controlled at 40% -60%. The normoxic groups were housed in a standard sea-level chamber (100.08 kPa, 20.9% O_2_) under identical temperature and humidity conditions. All mice had free access to standard laboratory food and water throughout the experiment. The duration of ligation or hypobaric hypoxia was 1,3, and 5 days. The maxillary bone, serum, and brain tissues were collected for further analysis. Prior to the experiment, all mice were screened for health status, and those with pre-existing severe periodontal disease or neurological disorders were excluded. Mice exhibiting swollen or bleeding gums, as well as abnormal weight loss, were excluded from the study and were humanely euthanized. Postoperative monitoring and care were provided to ensure that animals did not experience unnecessary pain during recovery. Behavioral tests were conducted in the following order: Morris water maze test, open field test, novel object recognition test, and elevated plus maze test. These tests were not performed on the same day to minimize potential interference between assessments.

### 2.2 Measurement of alveolar bone resorption

Microcomputed tomography (micro-CT) scanning was performed using SkyScan 1276 micro-CT (SkyScan 1276, Bruker, Germany) with 12 µm spatial resolution. Bone morphology analysis was conducted using the SkyScan 1276 Analysis System and Mimics Research software (version 21). Two-dimensional (2D) images of mandible scanning sections, reconstructed 3D microstructural images, and calculated structural indices were obtained. The linear distance from the cementoenamel junction (CEJ) to the alveolar bone crest (ABC) on the buccal side was measured.

### 2.3 Enzyme-linked immunosorbent assay

The blood samples collected from the mice were centrifuged at 4 °C and 3000 rpm for 15 minutes, and the supernatant was collected. Serum levels of interleukin-6 (IL-6, Cat: M6000, R&D Systems) and tumor necrosis factor-α (TNF-α, Cat: MTA00B, R&D Systems) were detected according to the product protocol.

### 2.4 Quantitative real-time PCR

Mouse tissues were homogenized in Trizol reagent (15596026, Invitrogen). Total RNA was extracted from the supernatant. The cDNA was synthesized using the Vazyme Reverse Transcription Kit (Cat: R333-01, Vazyme Biotech). To determine relative mRNA expression levels, qPCR was performed using the SYBR qPCR Master Mix reaction system. β-actin was used as the internal reference gene, and the sequences of the primers are listed in **Table 1**.

**Table 1:** Primer sequences used for real-time PCR.

Gene	Forward (5’–3’)	Reverse (5’–3’)
β-actin	ACTGTCGAGTCGCGTCCA	GTCATCCATGGCGAACTGGT
IL-6	AGTTGCCTTCTTGGGACTGA	TCCACGATTTCCCAGAGAAC
IL-1β	GCCCATCCTCTGTGACTCAT	AGGCCACAGGTATTTTGTCG
TNF-α	CGTCAGCCGATTTGCTATCT	CGGACTCCGCAAAGTCTAAG
MCP-1	CAGCTCTCTCTTCCTCCACC	TGGGATCATCTTGCTGGTGA
IL-8	CAGCTGCCTTAACCCCATCA	CTTGAGAAGTCCATGGCGAAA
KC	AGTAGAAGGGTGTTGTGCGA	CGTGCGTGTTGACCATACAA

### 2.5 HE staining

Brain tissues were dehydrated using a graded alcohol series. The brains were subsequently embedded in paraffin and sectioned. After deparaffinization and rehydration, the sections were stained with hematoxylin for 10 minutes, followed by differentiation in a differentiation solution. The sections were then washed in ammonia to restore a blue color. Next, they were stained with eosin. Finally, the samples were dehydrated again and sealed with neutral gum.

### 2.6 Nissl staining

The 5-μm paraffin sections were immersed in xylene for complete deparaffinization and subsequently hydrated in a gradient ethanol series. After washing with distilled water, the sections were stained with Nissl staining solution for 5 minutes. Following thorough rinsing with distilled water, the sections were air-dried and then sealed with drops of neutral gum.

### 2.7 Immunohistochemistry

Mice brains were fixed in 4% paraformaldehyde for 24 hours. Following gradient dehydration in sucrose solution, the brains were sectioned using a cryoslicer. Endogenous peroxidase activity was quenched with 10% H_2_O_2_. The sections were then permeabilized with 0.5% Triton X-100 and blocked with 5% BSA for 30 minutes. The sections were subsequently incubated overnight at 4°C with the following primary antibodies: Iba-1 (Cat: 019-19741, Wako), GFAP (Cat: MAB360, Millipore), CD16/32 (Cat: 553142, BD Biosciences), and CD206 (Cat: sc-58986, Santa Cruz). After washing with PBS, the sections were incubated for 1 hour in the dark with fluorescent secondary antibodies: Alexa Fluor 488-conjugated anti-rabbit antibody or Alexa Fluor 594-conjugated anti-mouse antibody (Life Sciences). Finally, the sections were sealed with a mixture of DAPI (Cat: ZLI-9557, ZSGB-BIO) and sealer.

### 2.8 Western blot

The samples were homogenized and lysed using RIPA lysis buffer supplemented with protease inhibitors and phosphatase inhibitors. Total proteins were extracted following centrifugation, and protein concentrations were quantified using a BCA kit. The proteins were separated by SDS polyacrylamide gel electrophoresis and transferred onto PVDF membranes, which were blocked with 5% skim milk for 1 hour. The target proteins were incubated with primary antibody STAT3 (Cat:3640S, Cell Signaling Technology), pSTAT3 (Cat:9143S, Cell Signaling Technology), TLR4 (Cat:sc-293072, Santa Cruz), MYD88 (Cat:4283S, Cell Signaling Technology), ZO-1 (Cat: ab96587, Abcam), Claudin5 (Cat: ab131259, Abcam), β-Actin (Cat: A2228, Sigma-Aldrich) at 4°C overnight, then washed by TBST. The membrane was incubated with HRP-conjugated secondary antibodies (Cat: 7076 and 7074, Cell Signaling Technology) for 2 hours, then washed by TBST and revealed with the ECL detection kit (Bio-Rad, Hercules, CA, USA).

### 2.9 Open field test

Open field test (OFT) was performed according to a reported experimental protocol (21). Briefly, each mouse was placed in the center of an open field (50 cm × 50 cm × 40 cm) and allowed to explore freely for 5 minutes. The bottom of the box was divided into 16 grids, and the central area was defined as four central grid areas, accounting for 25% of the total area. The test duration was 5 minutes, during which a camera recorded the distance traveled by the mice in the central area and the time spent there, using ANY-MAZE software for analysis.

### 2.10 Morris water maze test

Morris water maze test (MWMT) was carried out according to the reported experimental method with modifications (22) Morris water maze experiment device includes a circular pool (120 cm in diameter, 50 cm in height, divided into 4 quadrants), a platform, camera system, and analysis system. The circular platform (9 cm in diameter) is submerged 1 cm below the water level in the center of the fourth quadrant. The pool was dyed white with non-toxic titanium dioxide transparent agent, with water temperature maintained at 21°C ± 2°C. Additionally, curtains surrounding the pool feature distinct shapes, colors, and sizes corresponding to the four quadrants, serving as visual cues for the mice’s spatial memory. The Morris water maze experiment was divided into two phases: the learning trials and the probe test. During the learning trials, mice were given 60 seconds to explore the pool and located the hidden platform. If a mouse found the platform within the time limit, it was removed from the water. If the platform was not found, the mouse was guided to the platform and allowed to remain there for 10 seconds to memorize its location. After all mice completed training in one quadrant, they moved on to training in the next quadrant. In the probe test, the hidden platform was removed, and the mice were allowed to swim freely for 60 seconds. ANY-Maze software was used to record the swimming path and latency of the mice, reflecting their learning and memory abilities.

### 2.11 Elevated plus maze experiment

Elevated plus maze (EPM) experiment was conducted according to the reported experimental methods (23). The height of the maze platform is 50 cm, the length of the four arms is 35 cm, the height of the closed arm is 15 cm, and the width is 5 cm. The maze is a ‘cross’ shape, in which two arms are open arms and two arms are closed arms. Each mouse was placed in the center area facing an open arm and allowed to explore freely for 10 minutes, the time and number of entering the open arm or entering the closed arm were recorded with the ANY-MAZE software.

### 2.12 Novel object recognition experiment

Novel object recognition (NOR) experiment was performed as previously described (24). The test was carried out in a 50 cm × 50 cm × 40 cm plastic box under natural light conditions. The new object recognition experiment was divided into an adaptation phase, a familiarization phase, and a testing phase. During the adaptation phase, mice were placed in an empty box for 5 minutes to adapt to the environment. In the familiarization phase, two identical objects were placed in the box and the mice were allowed to explored for 15 minutes. During the testing period, one of the identical objects was replaced with a new object, the mice were explored the object for 10 minutes. The time spent exploring the new and old objects was recorded separately. Exploration is defined as a mouse sniffing or touching an object. The recognition index (RI) was calculated.

### 2.13 Statistical analysis

Experimental data were analyzed statistically using Graph pad Prism 8.0 software, data are presented as the mean ± standard error of the mean, The student’s t-test was used for the comparisons of two different groups from the experiments performed. The latency of MWMT was analyzed by two-way analysis of variance (ANOVA) followed by Turkey’s *post hoc* tests, and the other experiments were analyzed by one-way analysis of variance (ANOVA) followed by Turkey’s *post hoc* tests. Differences of P < 0.05 were statistically significant.

## 3 Result

### 3.1 Hypobaric hypoxia exposure aggravated periodontal and peripheral inflammation in periodontitis mice

**Figure 1:**
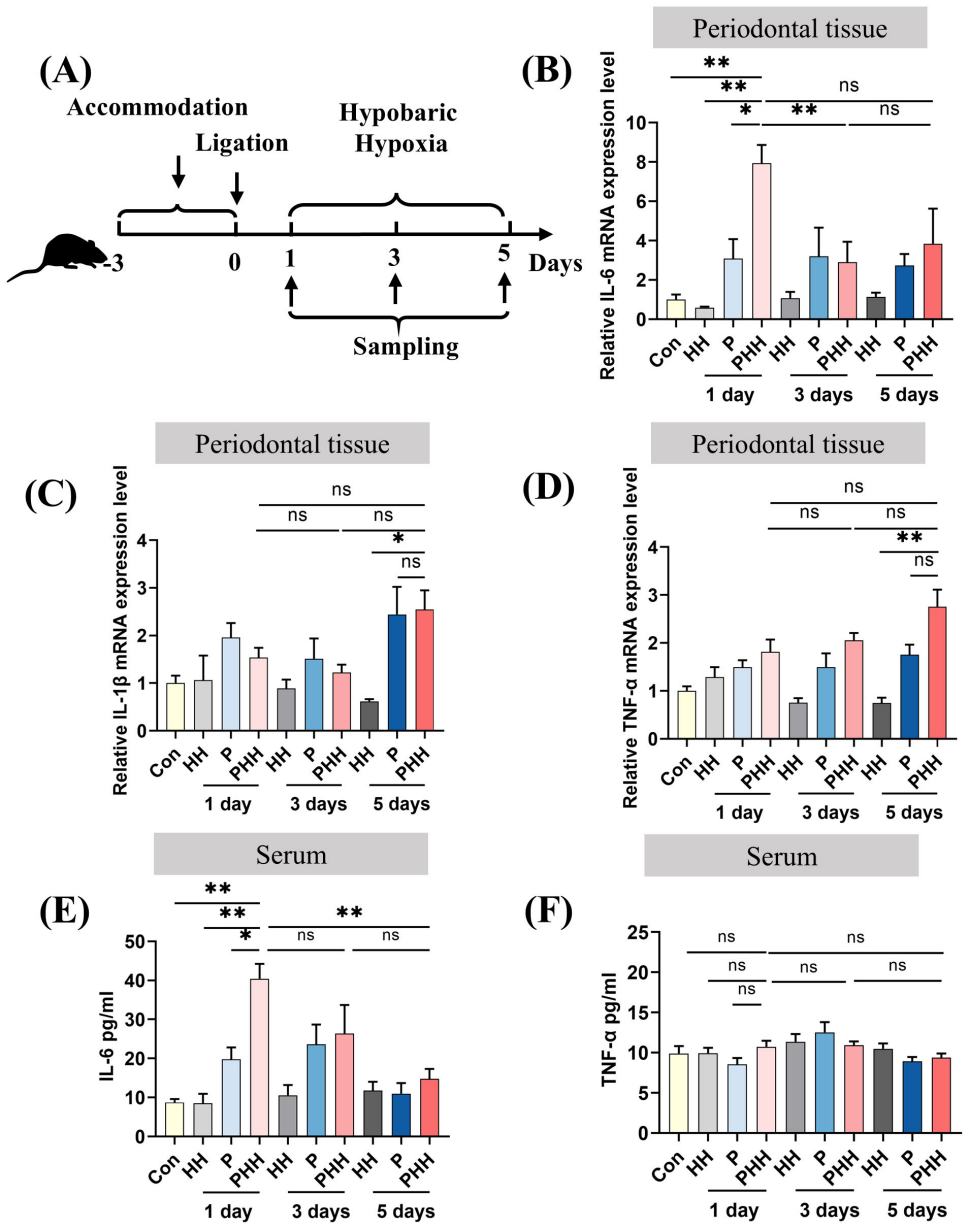
Hypobaric hypoxia exposure aggravated periodontal and peripheral inflammation in periodontitis mice. **(A)** Experiment scheme. **(B–D)** The mRNA expression of IL-6, IL-1β, and TNF-α in the periodontal tissue (n=3–6 animals per group), one-way ANOVA, *p<0.05, **p<0.01, ns, not significant. **(E, F)** The levels of IL-6 and TNF-α in the serum (n=4–6 animals per group), one-way ANOVA, *p<0.05, **p<0.01, ns, not significant. The results expressed as mean ± SEM, Con, control; HH, hypobaric hypoxia; P, periodontitis; PHH, periodontitis combined with hypobaric hypoxia.

To observe the effect of hypobaric hypoxia exposure over periodontal and peripheral inflammation, periodontitis mouse model was established first. As shown in **Supplementary Figure 1**, CEJ-ABC distances for the periodontitis group were significantly higher than those for control groups (281.8 ± 14.57 μm vs 203.0 ± 11.78 μm, p=0.0007), indicating the successful periodontitis mouse model. Mice with and without periodontitis were then placed in a hypobaric hypoxia chamber to simulate an environment equivalent to an altitude of 6000 meters. They were exposed to hypobaric hypoxia for 1 day, 3 days, or 5 days, respectively (**Figure 1A**). The levels of inflammatory factors in both periodontal tissues and serum were subsequently measured. The results revealed that, compared to other groups, the IL-6 mRNA level in periodontal tissues of periodontitis mice exposed to hypobaric hypoxia for 1 day (PHH 1-day group) was significantly upregulated, and then this increase was followed by a downregulation with prolonged exposure to hypobaric hypoxia (**Figure 1B**). As hypobaric hypoxia exposure time increased, the mRNA levels of IL-1β and TNF-α in periodontal tissues of periodontitis mice gradually rose, although no significant differences were observed compared to the periodontitis group (**Figures 1C, D**). The results from the ELISA assay showed that IL-6 levels in the serum from PHH 1-day group were increased significantly compared with the other three groups (**Figure 1E**). In contrast, there was no significant change in the serum level of inflammatory factor TNF-α in PHH 1-day group (**Figure 1F**). Collectively, these findings demonstrate that 1 day of hypobaric hypoxia exposure exacerbates both periodontal and peripheral inflammation in periodontitis model mice.

### 3.2 Hypobaric hypoxia exposure aggravated neuroinflammation in periodontitis mice

**Figure 2:**
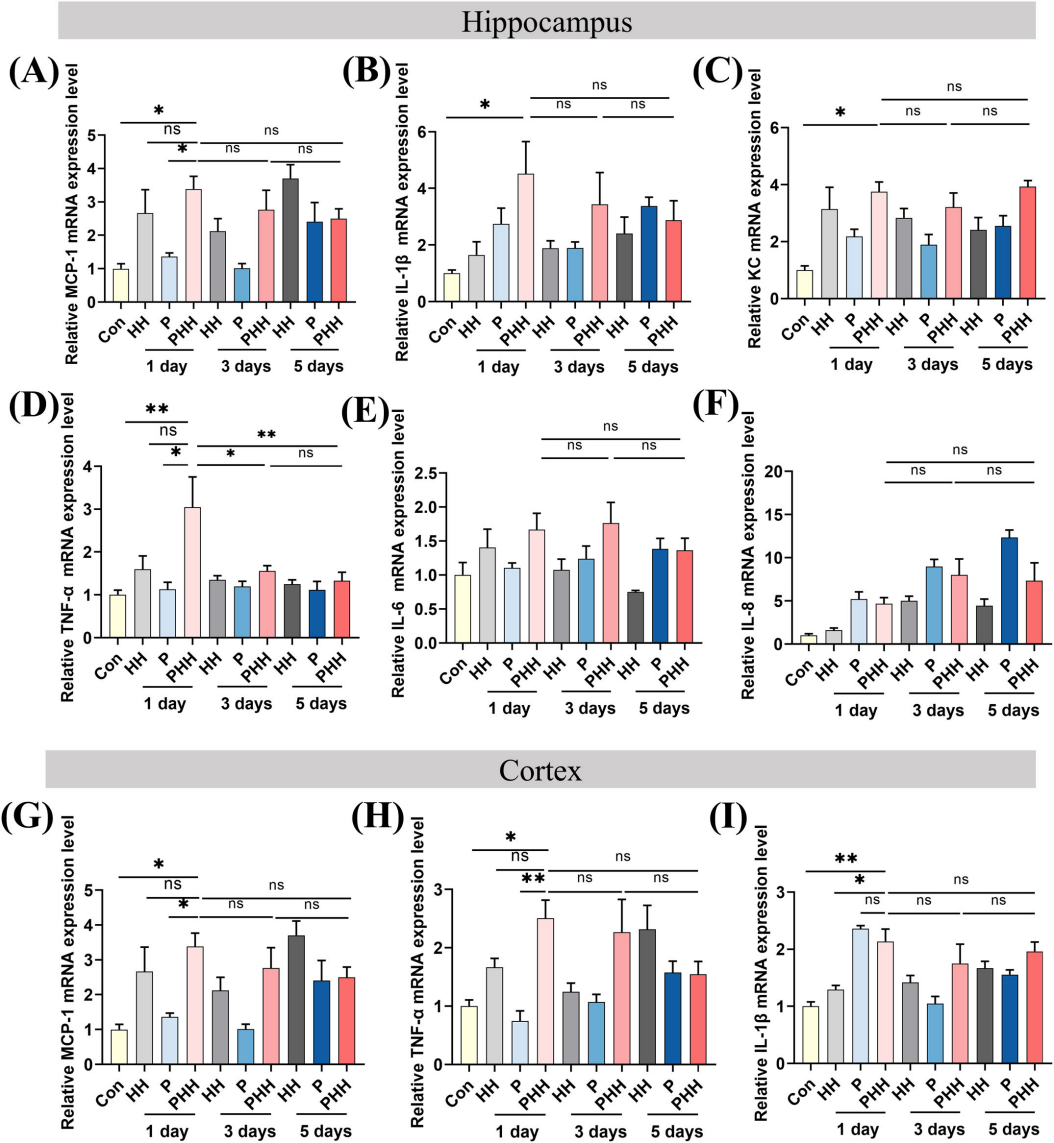
Hypobaric hypoxia exposure aggravated neuroinflammation in periodontitis mice. **(A–F)** The mRNA expression of IL-6, IL-1β, TNF-α, IL-8, MCP-1, KC in the hippocampus (n=3–5 animals per group), one-way ANOVA, *p<0.05, **p<0.01, ns, not significant. **(G–I)** The mRNA expression of IL-1β, TNF-α, and MCP-1 in the cortex (n=4–5 animals per group), one-way ANOVA, *p<0.05, **p<0.01, ns, not significant. The results are expressed as mean ± SEM, Con, control; HH, hypobaric hypoxia; P, periodontitis; PHH, periodontitis combined with hypobaric hypoxia.

To elucidate the influence of hypobaric hypoxia exposure on neuroinflammation in mice with periodontitis, the expression levels of various cytokines were assessed. Quantitative PCR analysis of hippocampal inflammatory cytokines revealed that the expression of the chemokine MCP-1 was significantly elevated in the PHH 1-day group compared to the Con or P 1-day groups (**Figure 2A**). The mRNA levels of IL-1β and KC in the PHH 1-day group did not differ significantly from those in the HH 1-day or P 1-day groups (**Figures 2B, C**). The inflammatory factor TNF-α was significantly upregulated in the PHH 1-day group compared to the Con or P 1-day groups (**Figure 2D**). However, the mRNA levels of IL-6 and IL-8 were comparable among the groups (**Figures 2E, F**).Additionally, quantitative PCR results for cortical inflammatory cytokines showed that the mRNA levels of MCP-1 and TNF-α were significantly higher in the PHH 1-day group compared to the Con or P 1-day groups, although no significant difference was observed compared to the HH 1-day group (**Figures 2G, H**). The mRNA level of IL-1β was significantly increased in the PHH 1-day group compared to the Con and HH 1-day groups (**Figure 2I**). Neuroinflammation can lead to impaired neural viability. Histological data from HE and Nissl staining assays indicated hippocampal tissue damage in the PHH 1-day group (**Supplementary Figure 2**). Collectively, these results demonstrate that acute exposure to hypobaric hypoxia exacerbates neuroinflammation in periodontitis mice.

### 3.3 Hypobaric hypoxia exposure polarized hippocampal microglia cells to M1 phenotype in periodontitis model mice

**Figure 3:**
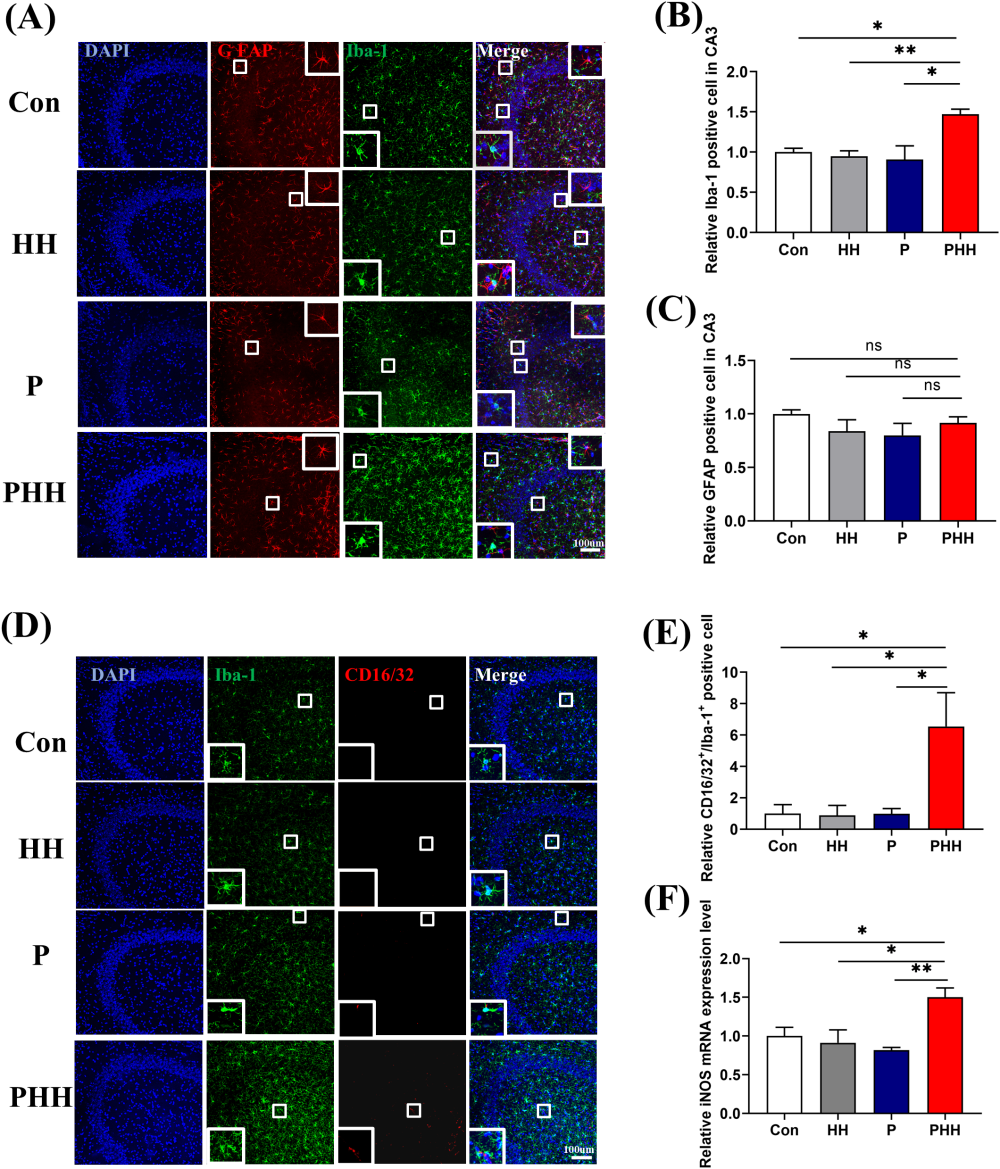
Hypobaric hypoxia exposure polarized hippocampal microglia cells to M1 phenotype in periodontitis model mice **(A)** Representative images of Iba-1 or GFAP positive cells in hippocampal CA3 region, scale bar is 100 μm. **(B, C)** Quantitative analysis of Iba-1 or GFAP positive cells in hippocampal CA3 region (n=3 animals per group), one-way ANOVA, *p<0.05, **p<0.01, ns, not significant. **(D)** Representative images of CD16/32 positive and Iba-1 positive cells in the hippocampal CA3 region, scale bar is 100 μm. **(E)** Statistical analysis of CD16/32 positive and Iba-1 positive cells in the hippocampal CA3 region (n=3 animals per group), one-way ANOVA, *p<0.05. **(F)** mRNA level of iNOS in the hippocampus (n=4 animals per group), one-way ANOVA, *p<0.05, **p<0.01. The results are expressed as mean ± SEM, Con, control; HH, hypobaric hypoxia 1day; P, periodontitis 1day; PHH, periodontitis combined with hypobaric hypoxia 1day.

To investigate whether microglia and astrocytes were involved in the process of hypobaric hypoxia exposure aggravating neuroinflammation in periodontitis model mice, their activation was examined. Immunofluorescence staining revealed that, compared to the other three groups, the number of Iba1-positive cells was significantly increased in the hippocampus of mice in the PHH group (**Figures 3A, B**). In contrast, the number of GFAP-positive cells did not differ significantly among the four groups (**Figure 3C**). Additionally, neither microglia nor astrocytes in the cortex exhibited significant activation (**Supplementary Figure 3**).

Activated microglia can polarize into M1 or M2 phenotypes, with M1 microglia expressing CD16/32, iNOS and M2 microglia expressing CD206, Arg-1. The results of hippocampal microglia polarization showed that the number of M1-type microglia was significantly elevated in the hippocampus of mice in the PHH group (**Figures 3D, E**), while the number of M2-type microglia remained unchanged (**Supplementary Figure 4**). Additionally, quantitative PCR analysis showed that, compared to the other three groups, the mRNA level of the M1 microglia marker iNOS was significantly upregulated in the PHH group mice (**Figure 3F**), although the mRNA level of the M2 microglia marker Arg-1 did not change (**Supplementary Figure 4**). All the data indicated that hypobaric hypoxia exposure polarized microglia into a pro-inflammatory phenotype in the hippocampus of periodontitis model mice and might enhancing neuroinflammation.

### 3.4 Hypobaric hypoxia exposure activated STAT3 signaling pathway in the brain of periodontitis mice

**Figure 4:**
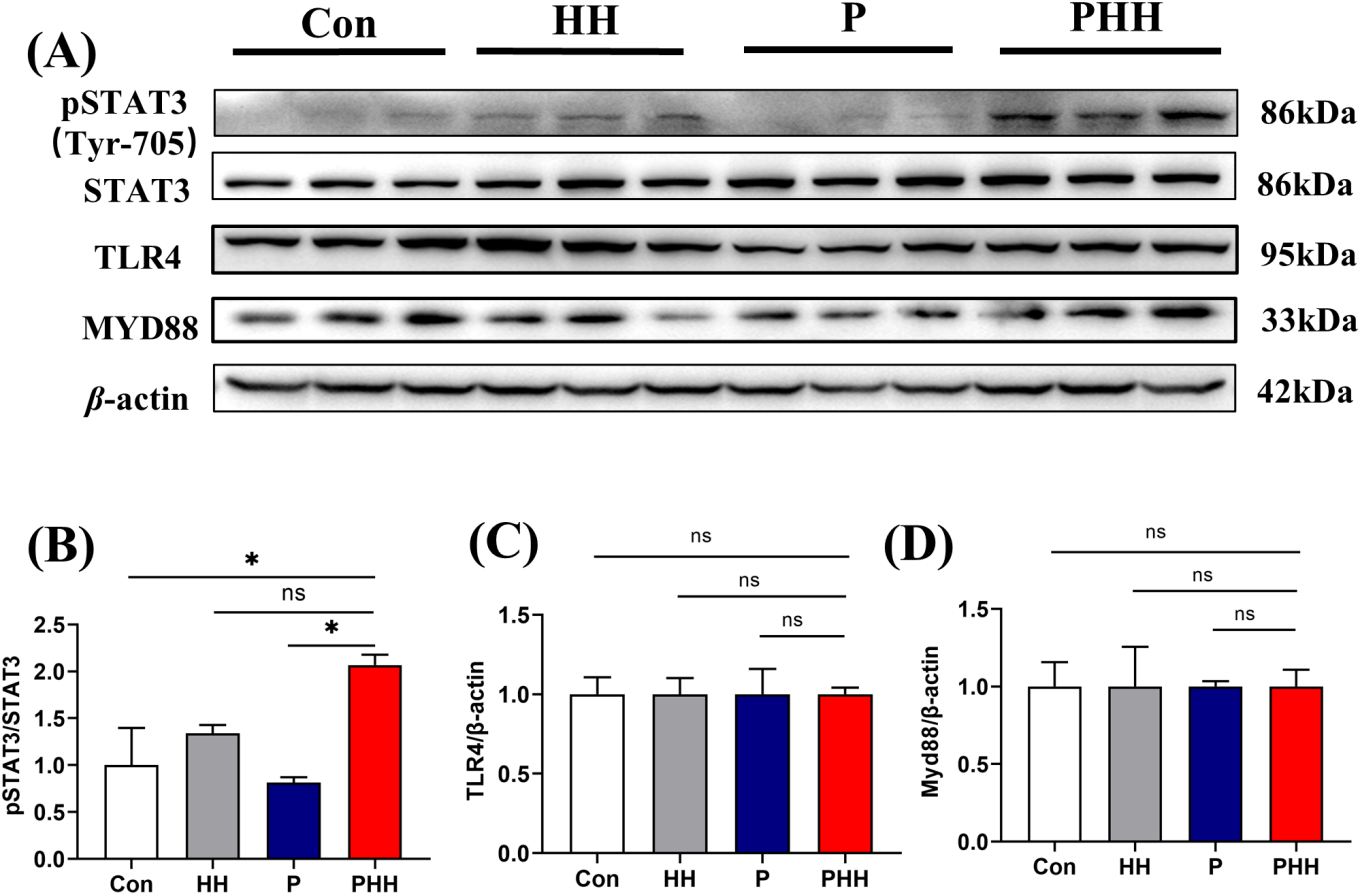
Hypobaric hypoxia exposure activated STAT3 signaling pathway in the brain of periodontitis mice. **(A)** Western blot images of pSTAT3, STAT3, TLR4, and MYD88 in the hippocampus (n=3 animals per group). **(B–D)** Quantitative analysis of pSTAT3, STAT3, TLR4, and MYD88 protein levels (n=3 animals per group), one-way ANOVA, * p < 0.05, ns, not statistically significant. The results were expressed as mean ± SEM, Con, control; HH, hypobaric hypoxia 1day; P, periodontitis 1day; PHH, periodontitis combined with hypobaric hypoxia 1day.

Signal transducer and activator of transcription (STAT) is one of the major regulatory elements in the production of inflammatory cytokines and signal molecules (25, 26). To further investigate the changes of inflammation signaling pathway in the process of hypobaric hypoxia exposure in periodontitis mice, protein expression of pSTAT3, STAT3, TLR4, and MYD88 in the hippocampus were examined. The results indicated that, compared to the Con or P 1-day groups, the expression of pSTAT3 was significantly upregulated in the PHH group mice, while the expression levels of STAT3, TLR4, and MYD88 remained unchanged (**Figures 4A–D**). These findings suggest that the exacerbation of neuroinflammation by hypobaric hypoxia exposure in periodontitis mice may be associated with the activation of the STAT3 signaling pathway.

### 3.5 STAT3 signaling pathway mediated aggravating neuroinflammation with hypobaric hypoxia exposure in periodontitis mice

**Figure 5:**
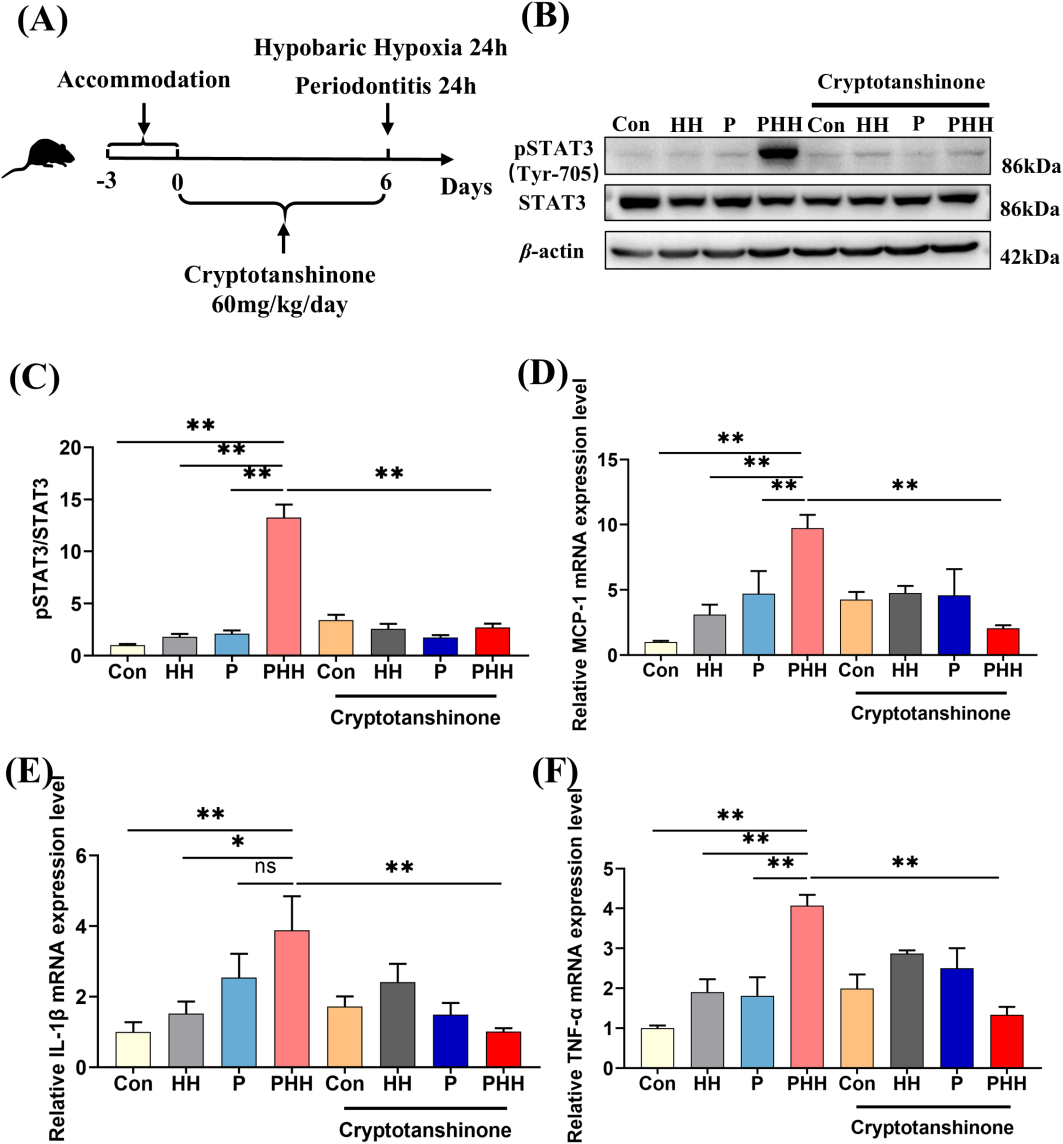
STAT3 signaling pathway mediated aggravating neuroinflammation with hypobaric hypoxia exposure in periodontitis mice. **(A)** Experiment scheme. **(B)** Western blot images of pSTAT3 and STAT3 in the hippocampus. **(C)** Quantitative analysis of pSTAT3 protein level (n=3 animals per group), one-way ANOVA, **p<0.01. **(D–F)** The mRNA levels of inflammatory factors IL-1β, TNF-α, and chemokine MCP-1 in the hippocampus (n=3–5 animals per group), one-way ANOVA, *p < 0.05, **p < 0.01, ns, not statistically significant. The results were expressed as mean ± SEM, Con, control; HH, hypobaric hypoxia 1day; P, periodontitis 1day; PHH, periodontitis combined with hypobaric hypoxia 1day.

To investigate the role of the STAT3 signaling pathway in the exacerbation of neuroinflammation by hypobaric hypoxia exposure in periodontitis mice, mice were orally administered the STAT3 signaling pathway inhibitor Cryptotanshinone at a dose of 60 mg/kg/day for 6 days. Subsequently, the mice were treated with periodontitis or hypobaric hypoxia for 24 hours (**Figure 5A**). The expression levels of STAT3 and pSTAT3 in the hippocampus was detected by western blot. The results showed that, compared to the other three groups, the expression of pSTAT3 in the hippocampus of mice in the PHH group was significantly upregulated (**Figures 5B, C**). However, oral administration of the STAT3 inhibitor significantly downregulated the expression of pSTAT3. Additionally, the levels of MCP-1, TNF-α, and IL-1β were significantly elevated in the PHH group mice but decreased following inhibition of the STAT3 signaling pathway (**Figures 5D–F**). These findings indicate that the activation of the STAT3 signaling pathway mediates the exacerbation of neuroinflammation in periodontitis model mice exposed to hypobaric hypoxia.

### 3.6 Hypobaric hypoxia exposure affected the learning and memory ability of periodontitis mice

**Figure 6:**
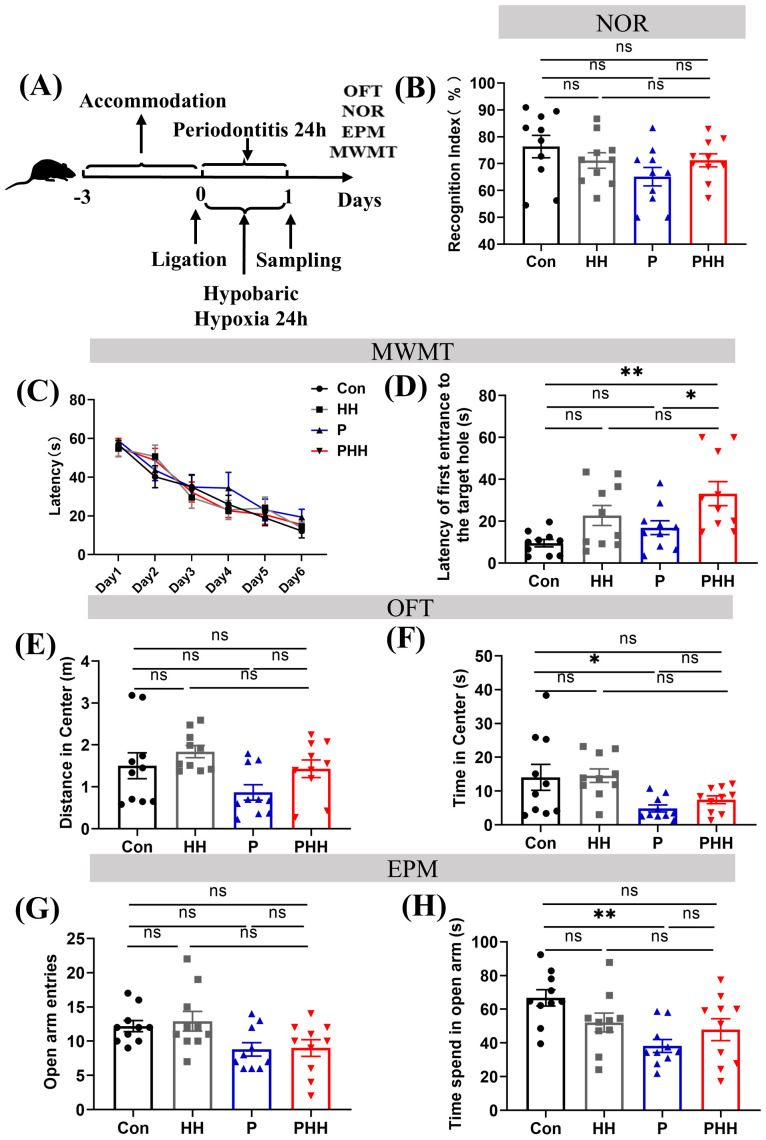
Hypobaric hypoxia exposure affected the learning and memory ability of periodontitis mice. **(A)** Mouse behavioral test process. **(B)** Mouse cognitive index in the novel object recognition experiment, RI = total sniffing time for the new object/(total sniffing time for the new object + total sniffing time for the old object) × 100% (n=10 animals per group), one-way ANOVA, ns, non-significant. **(C)** Learning curve of mice in water maze experiment (n=10 animals per group), two-way ANOVA. **(D)** Time to first reach the platform in water maze experiment (n=10 animals per group), one-way ANOVA, *p<0.05, **p<0.01, ns, non-significant. **(E)** Center movement distance in open field test (n=10 animals per group), one-way ANOVA, ns, non-significant. **(F)** Center movement time in open field test (n=10 animals per group), one-way ANOVA, *p<0.05, ns, non-significant. **(G)** Number of entries into the open arm in elevated plus maze experiment (n=10 animals per group), one-way ANOVA, ns, non-significant. **(H)** Open arm movement time in elevated plus maze experiment. (n=10 animals per group), one-way ANOVA, **p<0.01, ns, non-significant. The results are expressed as mean ± SEM, Con, control; HH, hypobaric hypoxia 1day; P, periodontitis 1day; PHH, periodontitis combined with hypobaric hypoxia 1day.

To evaluate whether hypobaric hypoxia affected the learning memory ability and anxiety-related behavior of periodontitis mice, several behavior experiments were carried out. The learning memory ability was tested by the novel object recognition (NOR) and Morris water maze test (MWMT). Besides, we evaluated the anxiety-related behavior of the mice by the open-field test (OFT) and elevated plus maze (EPM) (**Figure 6A**). In the novel object recognition (NOR), mice in the PHH group did not exhibit significant changes compared to other groups (**Figure 6B**). In the Morris water maze test (MWMT), the latency to reach the platform for the first time during the probe test was significantly increased in the PHH group compared to the control and periodontitis groups, although no significant difference was observed compared to the HH group (**Figures 6C, D**). In the open field test (OFT), periodontitis mice showed a significant reduction in the time spent in the center of the open field compared to control groups. However, this reduction did not further increase with hypobaric hypoxia exposure (**Figures 6E, F**). In the elevated plus maze (EPM), the time spent in the open arms was significantly decreased in periodontitis mice compared to the control group, indicating anxiety-like behaviors. However, this reduction did not further decrease with hypobaric hypoxia exposure (**Figures 6G, H**). Overall, these results suggest that periodontitis affects anxiety-related behaviors and cognitive abilities in mice. While hypobaric hypoxia exposure further impacts their spatial memory ability, it does not appear to have an additional effect on anxiety-related behaviors.

## 4 Discussion

**Figure 7:**
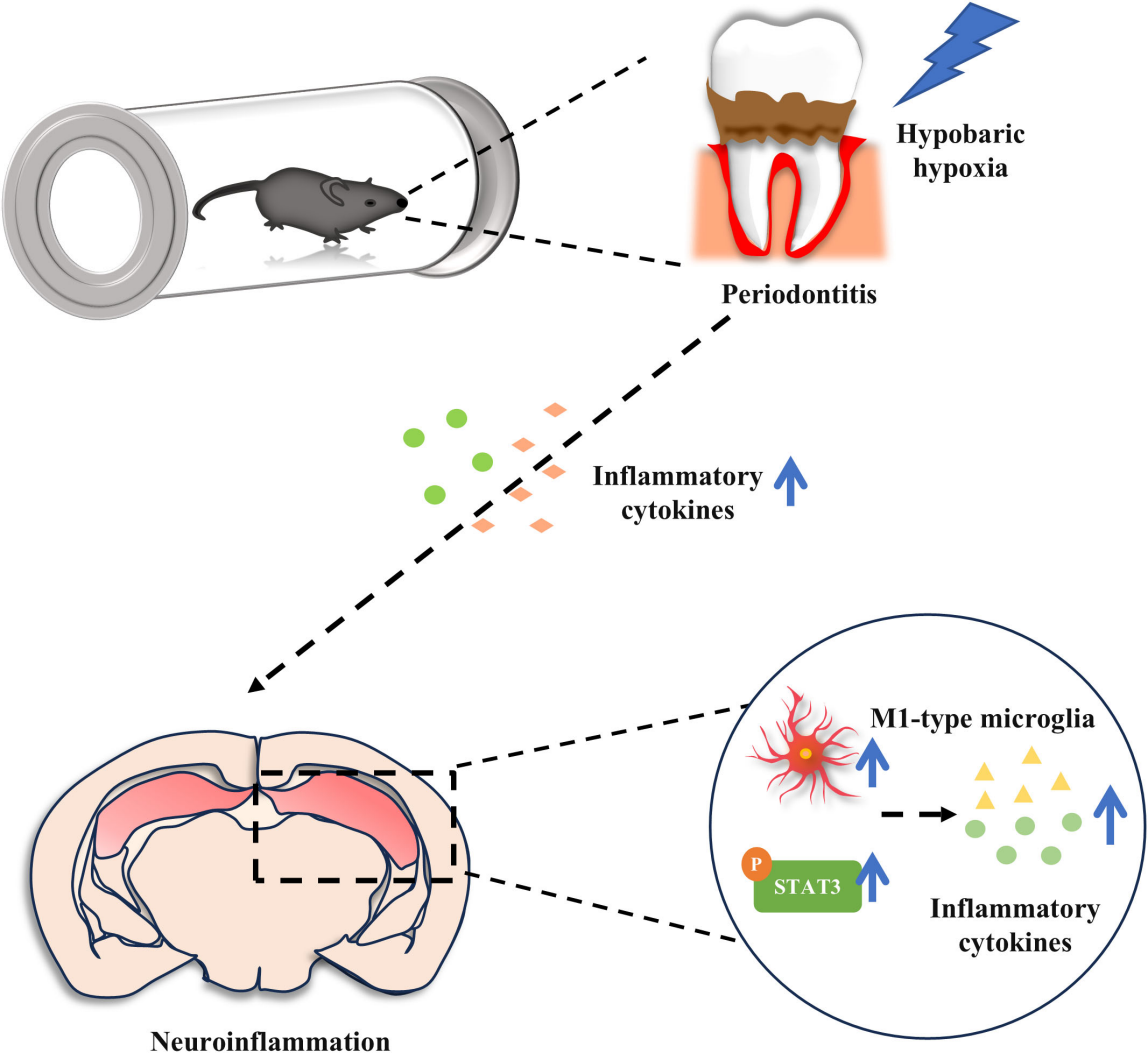
Acute hypobaric hypoxia exposure aggravates periodontal, peripheral inflammation and neuroinflammation in periodontitis mice. In addition, hypobaric hypoxia promotes M1 type microglial cell polarization and STAT3 activation in hippocampus of periodontitis mice.

In this study, we discovered that acute exposure to hypobaric hypoxia in mice with ligature-induced periodontitis triggered a more severe inflammatory response in both periodontal tissues and peripheral systems, which subsequently exacerbated neuroinflammation. Additionally, microglia in the hippocampus were significantly activated and polarized toward the M1 phenotype. Furthermore, the combination of periodontitis and hypobaric hypoxia activated the STAT3 signaling pathway, blocking this pathway attenuated the exacerbation of neuroinflammation induced by hypobaric hypoxia in periodontitis mice (**Figure 7**). Our findings provide novel insights into the complex interplay between periodontitis and neuroinflammation under the hypobaric hypoxia conditions typical of high-altitude environments.

It is worth noting that in this study, the duration of hypobaric hypoxia exposure had distinct effects on periodontitis, peripheral inflammation, and neuroinflammation in mice. Compared to the control group (Con), the HH 1-day group, and the P 1-day group, the level of IL-6 mRNA in the periodontal tissue of the PHH group was significantly upregulated. However, this combined effect diminished with prolonged exposure. Similarly, serum IL-6 levels in the PHH group initially increased significantly but then gradually decreased. In the hippocampus of PHH group mice, the levels of MCP-1 and TNF-α also increased but weakened over time. These findings suggest that 1 day of hypobaric hypoxia exposure has a more pronounced impact on periodontal inflammation, peripheral inflammation, and intracerebral neuroinflammation in periodontitis model mice compared to 3 or 5 days of exposure. This phenomenon may be attributed to the acute inflammatory response of local tissues to injury in the early stages (1 day) after periodontitis induction, during which IL-6, involved in the acute inflammatory response, reacts rapidly (27). Meanwhile, hypobaric hypoxia for 24 h in this process will cause a hypoxic stress response, further increase the level of IL-6 (28, 29), and aggravate brain inflammation through specific pathways. Additionally, related studies have shown that elevated IL-6 levels can promote alveolar bone resorption and aggravate periodontal inflammation (30), while hypoxia drives inflammatory mediators to worsen alveolar bone resorption (31). Therefore, it is speculated that compared with the periodontitis alone, the periodontitis with hypoxia will exhibit more severe alveolar bone resorption. Besides, the observed reduction in IL-6 levels after prolonged hypoxia exposure (3–5 days) may reflect compensatory anti-inflammatory mechanisms, as reported in literature (19). Additionally, tissues may gradually adapt to the hypoxic state, improving oxygen supply, leading to decreased IL-6 levels in periodontal tissues and serum, which in turn regulates the reduction of inflammation in the brain. These results indicate that hypobaric hypoxia applied during the acute phase of periodontitis exacerbates peripheral inflammation and promotes neuroinflammation, suggesting that patients with acute periodontitis should avoid high-altitude environments. Moreover, while IL-6 was significantly elevated in periodontal tissues and serum in the PHH group compared to other groups, no significant differences were observed in the brain. In contrast, IL-1β and TNF-α showed no significant changes in periodontal tissues and serum but exhibited significant differences in the brain. This discrepancy may be due to the rapid response characteristics of serum IL-6 in acute inflammatory responses (27), whereas IL-1β and TNF-α require more time or specific conditions to be significantly upregulated. Additionally, brain IL-6 is primarily secreted by astrocytes and microglia, and the PHH model may not trigger the IL-6-specific pathways in these cells. These differences highlight the complexity of the regulatory mechanisms and physiological functions of different cytokines. Similar complex changes in cytokines were observed in a one-week periodontitis mouse experiment. Serum IL-6 levels increased, while IL-1β and TNF-α did not change significantly. In the hippocampus, IL-1β levels increased, while IL-6 and TNF-α remained unchanged (19).

Microglia and astrocytes are the primary immune cells in the brain and a major source of pro-inflammatory cytokines (32). Immunofluorescence results revealed that, following the combination of periodontitis and hypobaric hypoxia, the number of hippocampal microglia significantly increased and these cells were activated, whereas astrocytes showed no significant changes. This may be attributed to microglia being more sensitive to hypobaric hypoxia stimulation compared to astrocytes in terms of inflammatory response. Under hypobaric hypoxic conditions, microglia are first activated and release a series of cytokines and chemokines to cope with the damage caused by hypobaric hypoxia (33). Furthermore, in the hippocampus of mice subjected to periodontitis combined with hypobaric hypoxia, activated microglia were polarized toward the M1 phenotype, thereby enhancing their pro-inflammatory effects. Compared with the hippocampus, the cortical microglia were not significantly activated. This may be due to the fact that hippocampal microglia are more easily activated than cortical microglia under a variety of conditions (34–36). Additionally, Nissl staining and HE staining showed that the hippocampus was damaged, while the cortex did not change significantly (**Supplementary Figure 3**), which may be due to the fact that the hippocampus is more sensitive to hypobaric hypoxia than the cortex (37–41). Therefore, the activation and release of chemokines and inflammatory factors by hippocampal microglia in ligature-induced periodontitis may be a key factor leading to neuroinflammation.

Studies have shown that ligature-induced periodontitis activates the STAT3 signaling pathway leading to neuroinflammation in the brain of rats (9). Moreover, STAT3 inhibitors have been shown to significantly reduce the expression of IL-6 and IL-1β in the brain (11). In this study, exposure to hypobaric hypoxia significantly enhanced the activation of the STAT3 signaling pathway in periodontitis mice. Upon inhibition of the STAT3 signaling pathway, the levels of MCP-1, TNF-α, and IL-1β in the brain were markedly reduced. This suggests that the STAT3 signaling pathway exerts a pro-inflammatory effect in the process of hypobaric hypoxia exposure exacerbating neuroinflammation in periodontitis model mice. However, it cannot be denied that other signaling pathways related to hypoxia or inflammation may also play an important role in this process, such as the NF-κB signaling pathway related to hypobaric hypoxia and periodontitis. It has been reported that LPS and hypoxic treatment activate the NF-κB signaling pathway leading to neuroinflammation (33, 42).

In addition, neurobehavioral experiments were conducted in this study to explore the effects of hypobaric hypoxia exposure on the emotional state and spatial cognitive ability of periodontitis model mice. However, the results were not as clear-cut as anticipated. Several potential reasons may account for this: Firstly, there may have been mutual interference between different behavioural experiments, which could have confounded the outcomes. Secondly, due to the limitations of our experimental setup, behavioural experiments could not be conducted within a hypobaric hypoxia environment. Mice were reoxygenated during the process of being removed from the hypobaric hypoxia chamber for testing, which likely had a significant impact on the results. In the open field experiment and the elevated plus maze, compared with the control group, the central time and open arm time of the mice in the open field of the P group were significantly shortened, and the mice showed anxiety-like behaviours. However, the anxiety of the PHH group was not further aggravated. This may be attributed to the heightened excitability of the mice during reoxygenation, which could have masked the effects of hypobaric hypoxia on anxiety. Lastly, in this experiment, the level of inflammation caused by the combination of periodontitis and hypobaric hypoxia was relatively lower compared to other inflammatory models. It remains unclear whether this level of inflammation was sufficient to impact the neurobehavior of mice. Therefore, further experimental design and exploration are needed to elucidate these effects more clearly.

Our study acknowledges several limitations. Firstly, our investigation focused solely on the effects of acute hypobaric hypoxia exposure (1-day) on periodontitis. This short-term exposure may not accurately represent the chronic inflammatory responses observed under prolonged high-altitude conditions. Future research should explore the impacts of hypobaric hypoxia over extended periods to provide a more comprehensive understanding of its effects on peripheral inflammation. Secondly, the absence of bacterial application, such as a challenge with Porphyromonas gingivalis, restricts the translational relevance of our findings to human periodontitis. The inclusion of such bacterial challenges is crucial for mimicking the microbial etiology of periodontal disease in humans. Future studies should employ diverse mouse periodontitis models incorporating specific bacterial challenges to better replicate the varied clinical presentations of human periodontitis.

In summary, we found that hypobaric hypoxia exposure aggravated systemic inflammation and even promoted neuroinflammation in ligature-induced periodontitis mice, and the STAT3 signaling pathway may play an important role in inducing and amplifying neuroinflammation.

## 5 Conclusion

In conclusion, this study found that acute hypobaric hypoxia exposure aggravated periodontal inflammation, systemic inflammation, and neuroinflammation in periodontitis mice. The relationship between periodontitis and neuroinflammation in a high-altitude hypobaric hypoxia environment was proposed for the first time. Further research may provide new targets for the prevention and treatment of high-altitude brain injury in patients with periodontitis.

## Data Availability

The original contributions presented in the study are included in the article/**Supplementary Material**. Further inquiries can be directed to the corresponding authors.
