# Supplementary figures and images for: Hypobaric hypoxia aggravates neuroinflammation in ligature-induced periodontitis mice via the STAT3 signaling pathway
Source: Front Immunol. 2025 Jul 29;16:1600035. doi: 10.3389/fimmu.2025.1600035 (PMC12339329; doi:10.3389/fimmu.2025.1600035)

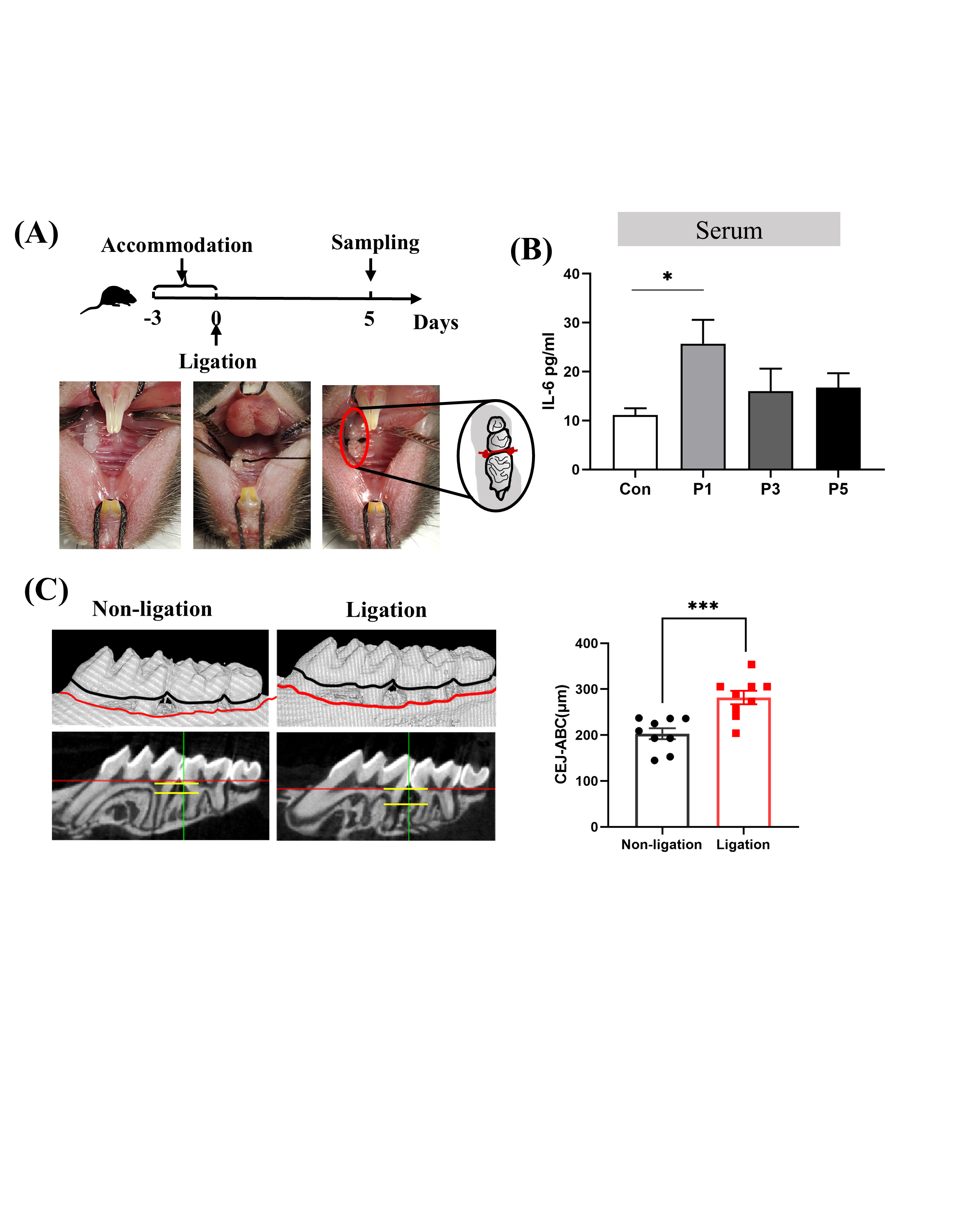

Supplement: Supplementary file 1 [file Image1.tif]

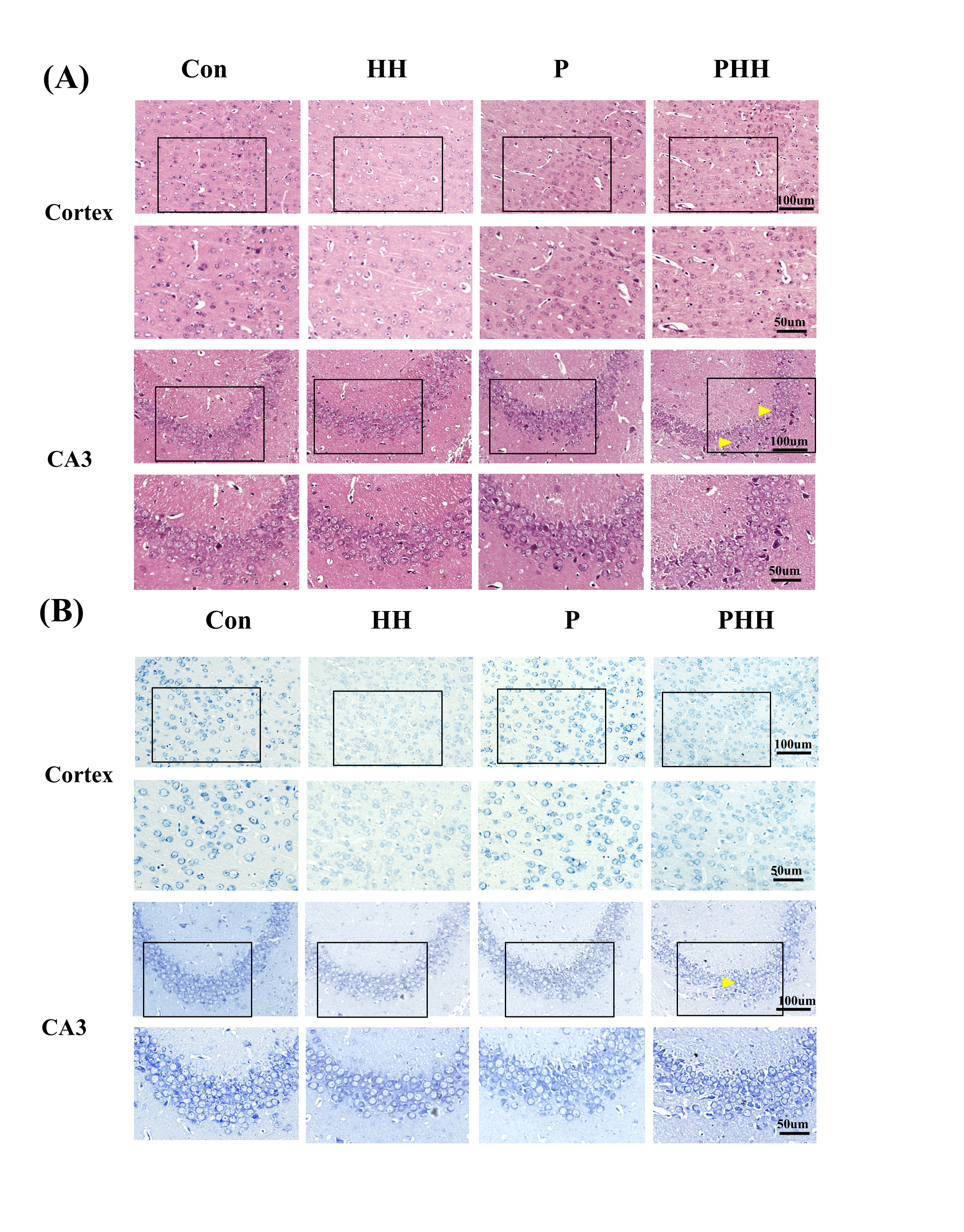

Supplement: Supplementary file 2 [file Image2.tif]

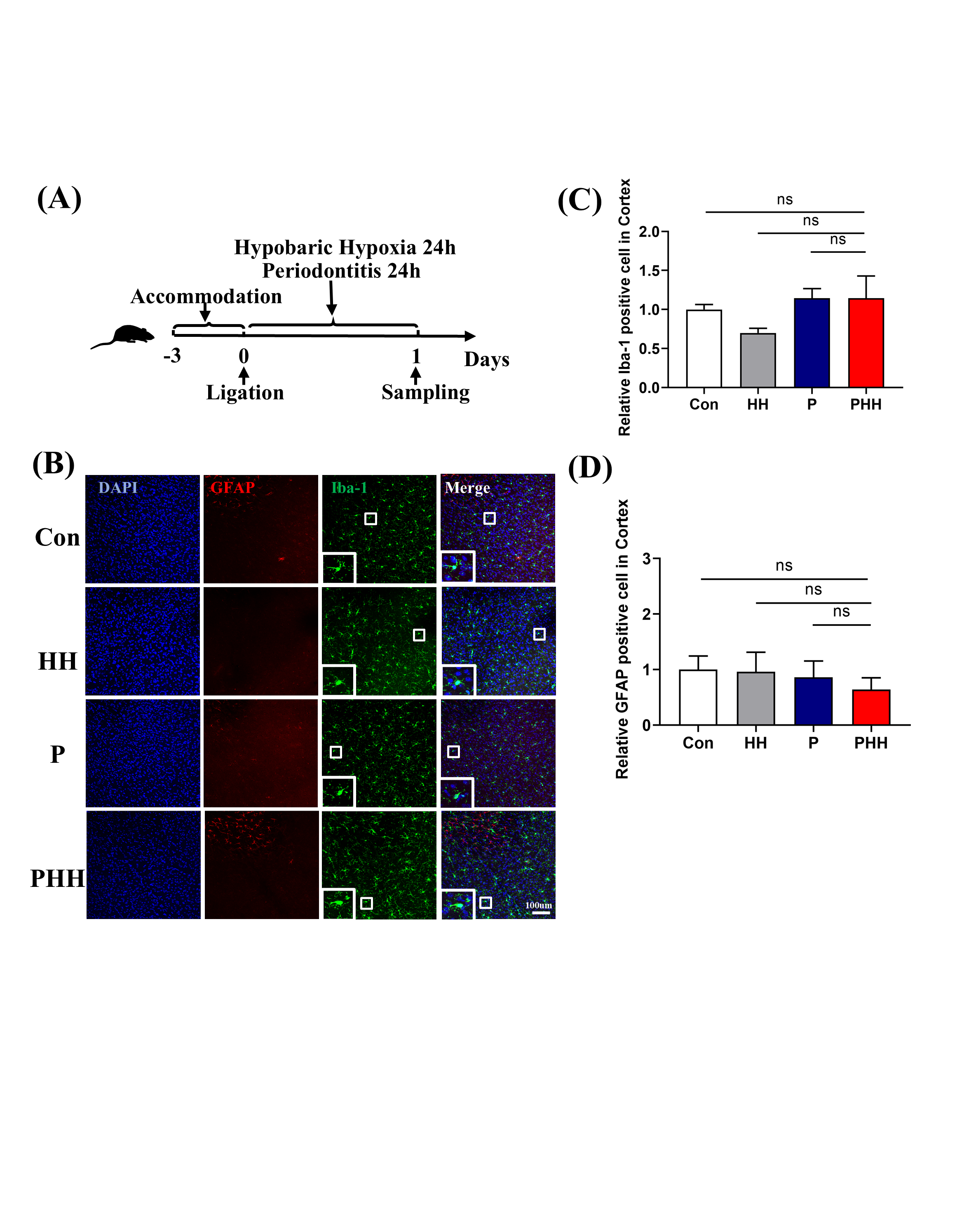

Supplement: Supplementary file 3 [file Image3.tif]

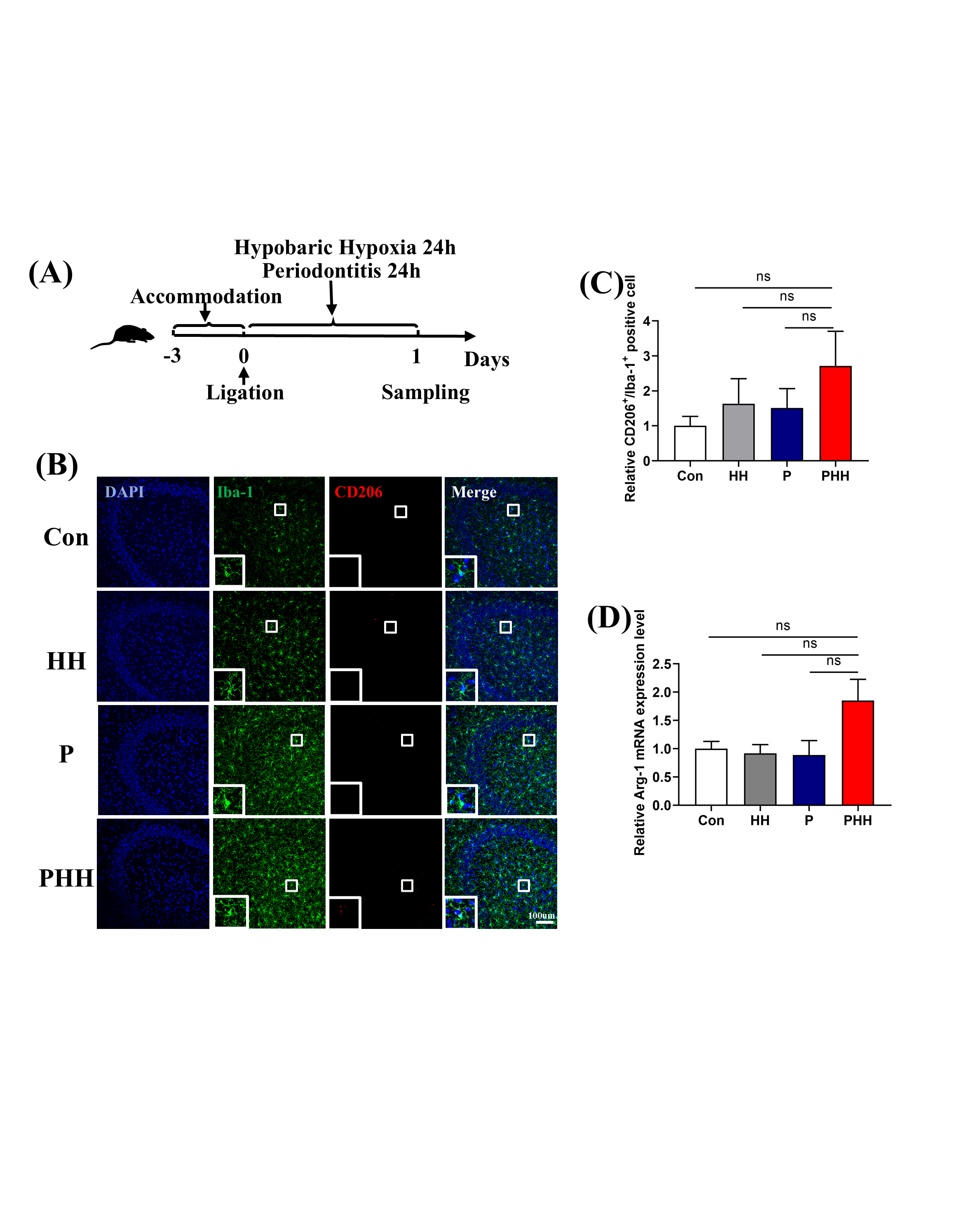

Supplement: Supplementary file 4 [file Image4.tif]
