# Supplementary material for: Hypobaric hypoxia aggravates neuroinflammation in ligature-induced periodontitis mice via the STAT3 signaling pathway
Source: Front Immunol. 2025 Jul 29;16:1600035. doi: 10.3389/fimmu.2025.1600035 (PMC12339329; doi:10.3389/fimmu.2025.1600035)
Supplement: Supplementary file 5 [file Table1.docx]

Supplementary Material

# Supplementary Figures and Tables

## Supplementary Figures

Supplementary Figure 1. Establishment of a periodontitis mouse model. (A) Experiment scheme and the process of establishing a periodontitis mouse model. (B) The mRNA expression of IL-6 in the serum after 1, 3, and 5 days of periodontitis (n=6-7 animals per group), one-way ANOVA, *p<0.05, ns, non-significant. (C) Two-dimensional and three-dimensional sagittal micro-CT scans were performed on the maxillary model side and the control side of mice with periodontitis for 5 days. The distance from the cement enamel junction (CEJ) to the alveolar bone crest (ABC) was measured. (n=9 animals per group), the student’s t-test, **p<0.01, ns, non-significant. The results are expressed as mean ± SEM.

**Supplementary Figure 2.** Hypobaric hypoxic exposure aggravated hippocampal tissue damage in periodontitis mice. (A) Representative photomicrographs of HE staining in the cortex and hippocampus (n=3 animals per group), scale bar=100μm or 50μm. (B) Representative photomicrographs of Nissl staining in the cortex and hippocampus (n=3 animals per group), scale bar=100μm or 50μm. Con: control, HH: hypobaric hypoxia 1day, P: periodontitis 1day, PHH: periodontitis combined with hypobaric hypoxia 1day.

**Supplementary Figure 3.** Hypobaric hypoxia exposure did not activate cortical glia cells in periodontitis model mice. (A) Experiment scheme. (B) Representative images of Iba-1 positive and GFAP positive cells in the cortex (n=3-4 animals per group), one-way ANOVA, ns, non-significant. (C) Quantitative analysis of Iba-1 positive cells in the cortex (n=3-4 animals per group), one-way ANOVA, ns, non-significant. (D) Quantitative analysis of GFAP positive cells in the cortex (n=3-4 animals per group), one-way ANOVA, ns, non-significant. The results are expressed as mean ± SEM. Con: control, HH: hypobaric hypoxia1day, P: periodontitis 1day, PHH: periodontitis combined with hypobaric hypoxia 1day.

**Supplementary Figure 4.** Hypobaric hypoxia exposure did not polarize hippocampal microglia into M2 phenotype in periodontitis model mice. (A) Experiment scheme. (B) Representative images of CD206 positive cells and Iba-1 positive cells in the hippocampal CA3 region, scale bar=100 μm. (C) Statistical analysis of CD206 positive cells and Iba-1 positive cells in the hippocampal CA3 region (n=3 animals per group), one-way ANOVA, ns, non-significant. (D) mRNA level of Arg-1in the hippocampus (n=4 animals per group), one-way ANOVA, ns, non-significant. The results are expressed as mean ± SEM, Con: control, HH: hypobaric hypoxia 1day, P: periodontitis 1day, PHH: periodontitis combined with hypobaric hypoxia for 1 day.
